# Impact of social determinants of health on post hospital care following orthopaedic polytrauma

**DOI:** 10.1007/s00590-026-04939-z

**Published:** 2026-09-08

**Authors:** David Dallas-Orr, Jessica Valdez, Shannon Tse, Mark A. Lee, Megan R. Terle, Gillian L. S. Soles, Ellen P. Fitzpatrick, Sean T. Campbell, Barton L. Wise, Augustine M. Saiz

**Affiliations:** 1 Department of Orthopaedic Surgery, University of California Davis Health System, Sacramento, USA; 2 School of Medicine, University of California Davis, Sacramento, USA

**Keywords:** Orthopaedic polytrauma, Social determinants of health, Area deprivation index, Socioeconomic distress

## Abstract

**Purpose:**

To determine whether community socioeconomic distress, measured by the Area Deprivation Index (ADI), is associated with worse short-term clinical outcomes in orthopaedic polytrauma patients treated at an academic level I trauma center.

**Methods:**

We performed a retrospective cohort study of polytrauma patients treated for orthopaedic injuries at an academic level I trauma center (2014–2019). State ADI scores were used to classify patients as residing in a distressed community (ADI > 8; DC) versus non-distressed community (NDC). Collected variables included demographics, injury characteristics including the Injury Severity Score (ISS), insurance status, and outcomes. Primary outcomes were malunion, nonunion, and unplanned return to the operating room; secondary outcomes included disposition at discharge and follow-up duration.

**Results:**

Among 348 patients, 193 (55.5%) resided in DC. Compared with NDC, a higher proportion of DC patients identified as Black or American Indian/Pacific Islander (*p* < 0.002), and DC patients more frequently had a prior smoking history (57.4%) and psychiatric illness (19.7%; both *p* < 0.001) and carried public insurance (*p* = 0.003). Mechanisms of injury differed (*p* = 0.045). Mean ISS was similar between DC and NDC (18.8 vs. 19.8; *p* = 0.435), and patients from DC were less likely to be discharged home (48.9 vs. 62.0%; *p* = 0.021). Rates of malunion, nonunion, and reoperation were similar between groups.

**Conclusions:**

Our findings indicate that orthopaedic polytrauma patients from distressed communities are disproportionately affected by factors that complicate recovery, including smoking history, psychiatric comorbidities, and insurance status. Despite these associations, ADI was not associated with short-term outcomes in our polytrauma patient population.

## Introduction

Unintentional injuries are the third leading cause of death in the United States and the leading cause of death of people under the age of 45 [1]. Many studies have evaluated the mechanisms and demographics of trauma patients [2–4]. However, there are a limited number of studies evaluating the social determinants of health in polytrauma patients. Polytrauma can be defined using the Berlin polytrauma definition (BPD), which specifies polytrauma patients to have an Abbreviated Injury Score (AIS) of ≥ 3 in at least 2 different areas of the body, along with meeting at least one of the defined physiologic parameters (hypotension, old age, acidosis, coagulopathy, and decreased consciousness) [5]. An observational study evaluating 300,649 acute trauma admissions from the Dutch National Trauma Register, showed that the BPD was able to improve the accuracy of identifying patients with higher resources needs and mortality [6].

In 2013–2014, there was 1,556,920 orthopedic trauma patients admitted to hospitals in the U.S. and 530,215 of those patients also had concurring polytrauma [7]. Although there is variation in the criteria used to define polytrauma, these statistics are consistent with a more recent single center study that showed that polytrauma represents approximately 25% of major trauma admissions [8]. Lastly, the Injury Severity Score (ISS) which is derived from the AIS. It is calculated by taking the sum of squares of the AIS scores of the three most severely injured body regions [9]. There are several modified versions of the ISS, however the ISS is the most used anatomic trauma severity scoring system and has been shown to have the best predictive capacity for trauma patient admission to the intensive care unit [10]. 

Although access to trauma care has increased across the United States, significant health disparities have been reported for vulnerable populations [11, 12]. The causes of health disparities among trauma patients have been reviewed by Bradley et al. [13] Race, ethnicity, socioeconomic status, and access to healthcare have been associated with inadequate pain management, reduced post discharge care, and increased mortality. There are many metrics to quantify the social determinants of health to allow for more equitable healthcare delivery [14]. The Area Deprivation Index (ADI) is one metric that helps determine the level of socioeconomic disadvantage in certain regions and it is based on neighborhood level census data [15, 16]. Higher ADI values signify higher levels of socioeconomic disadvantage. ADI values are available to evaluated communities at the state or national level, with the values being presented in deciles and percentiles respectively. This study’s objective was to compare the demographics, injury characteristics and clinical outcomes of polytrauma patients based on socioeconomic status as measured by ADI.

## Patients and methods

After obtaining institutional review board approval, a retrospective chart review of polytrauma patients (ISS > 20 or BPD) was performed at a Level 1 Trauma Center between 2014 and 2019. The ISS was the trauma severity scoring used in this study because it is the most widely used anatomical trauma severity scoring system. The ISS was recorded for each patient and compared between the two groups. Patient demographics, injury, and outcome variables were recorded. Based on zip code, state ADI scores were collected and used to determine differences in post-hospital care, including considering those with an ADI > 8 as disadvantaged from living in a distressed community [Group 1: Distressed Community (DC); Group 2: Non-Distressed Community (NDC)]. Eight patients had addresses for which a state ADI score could not be assigned (out-of-state or group-quarters residence); comparisons of ISS, length of stay, and discharge disposition excluded these patients (NDC *n* = 150; DC *n* = 190). Outcome variables included malunion, nonunion, unplanned return to the operating room (UROR), final disposition, and total follow-up. Malunion and nonunion were identified from the diagnoses documented in the medical record by the treating team and confirmed by review of the radiographs by orthopaedic surgery residents. Malunion was defined as healing of a fracture in an abnormal position that leads to impaired function of the bone or limb. Nonunion was defined as no visible sign of healing for 3 months with a minimum time of 9 months since the fracture. The criteria for UROR were as follows: return to the operating room within 24 h of the index operation due to a cause that is likely related to the index operation, no documentation of any planned or staged surgery.

Statistical analyses were conducted using SPSS (IBM) and a *p*-value of < 0.05 was used to determine statistical significance. Descriptive statistics were obtained to evaluate demographic, socioeconomic, and outcome variables. Chi-square tests, Fisher exact tests, and t-tests were used to evaluate potential differences between the two groups. Odds ratios (OR) with 95% confidence intervals (CI) were calculated for key binary characteristics.

## Results

This study evaluated 348 patients that met polytrauma criteria, with 193 of these patients (55.5%) residing in DC (ADI > 8). The cohort included 228 (65.5%) males and 120 (34.5%) females, and the average BMI of the cohort was 27.4 (± 6.49). Most patients identified as White (27.6%) or other race (27.6%) and identified as having non-Hispanic or Latino ethnicity (80.1%). The mean Charlson Comorbidity Index was 1.42 ± 1.93, indicating a moderate level of comorbidity throughout the cohort. Lastly, most patients had private insurance (43.5%), followed by Medi-Cal (23.2%), and Medicare (22.1%) (Table 1).

**Table 1:** Demographics and comorbidities stratified by area deprivation index

	Overall	NDC ADI < 8	DC ADI > 8	*p*-value
Patients, n (%)	348	155 (44.5)	193 (55.4)	
Age, mean ± SD	45.9 ± 19.3	46.7 ± 18.6	45.2 ± 19.9	0.491
Sex, n (%)				
Female	120 (34.5)	48 (30.9)	72 (37.3)	0.216
Male	228 (65.5)	107 (69.0)	121 (62.7)	
Race, n (%)				
African American	30 (8.8)	9 (6)	21 (10.9)	**0.002**
Asian	26 (7.62)	20 (13.3)	6 (3.14)	
Other	94 (27.6)	32 (21.3)	62 (32.5)	
White	191 (27.6)	89 (59.3)	102 (53.4)	
Ethnicity, n (%)				
Hispanic or Latino	68 (19.9)	22 (14.5)	46 (25.3)	0.076
Not Hispanic or Latino	273 (80.1)	130 (85.5)	143 (75.7)	
Insurance, n (%)				
Private	124 (43.5)	72 (54.6)	52 (33.9)	**0.003**
MediCal	66 (23.2)	23 (17.4)	43 (28.1)	
Medicare	63 (22.1)	28 (21.2)	35 (22.9)	
California Children’s Services	10 (3.51)	0 (0)	10 (6.54)	
Veterans Affairs	8 (2.81)	1 (0.76)	7 (4.58)	
No insurance	14 (4.91)	8 (6.06)	6 (3.91)	
BMI, mean ± SD	27.4 ± 6.49	27.1 ± 5.86	27.7 ± 7.13	0.359
CCI, n (%)	1.42 ± 1.93	1.39 ± 1.93	1.44 ± 1.94	0.842
Smokers, n (%)	161 (46.3)	56 (36.8)	105 (55.6)	**0.002**
Psychiatric history, n (%)	50 (14.4)	12 (7.74)	38 (20.1)	**0.002**

Note: Bolded *p*-values indicate statistical significance (*p* < 0.05). Abbreviations: ADI: Area Deprivation Index; BMI: Body Mass Index; CCI: Charlson Comorbidity Index; DC: Distressed Community; NDC: Non-Distressed Community; SD: Standard Deviation

The injury characteristics and outcome variables for the entire cohort are reported in Table 2. The mean ISS was 19.4 ± 12.6 (median 18, range 0–66), and 141 patients (40.5%) had an ISS greater than 20. The three most common mechanisms of injuries were due to motor vehicle crash (MVC; 33.1%), ground-level fall (16.7%) and auto vs. pedestrian injury (13.3%) (Table 2). A total of 82 (23.6%) of these injuries were classified as emergent. Overall, the average length of stay was 24.0 days (SD = 130). Additionally, most patients were discharged home (54.6%) (Table 2). Overall, very few patients experienced malunion (0.86%) and few experienced nonunion (2.87%). Reoperation was required in 60 patients (17.2%) and patients had frequent follow-up with an average of 233 ± 385 days (Table 2).

**Table 2:** Injury characteristics and outcome variables stratified by area deprivation index

	Overall	NDC ADI < 8	DC ADI > 8	*p*-value
Patients, n	348	155 (44.5)	193 (55.4)	
ISS, mean ± SD	19.4 ± 12.6	19.8 ± 13.2	18.8 ± 11.9	0.435
Mechanism of Injury, n (%)				
Motor vehicle crash	115 (33.1)	47 (30.5)	68 (35.2)	**0.045**
Motorcycle crash	39 (11.2)	15 (9.74)	24 (12.4)	
Ground level fall	58 (16.7)	28 (18.2)	30 (15.5)	
Fall from height	37 (10.6)	18 (11.7)	19 (9.84)	
Auto versus pedestrian	46 (13.3)	18 (11.7)	28 (14.5)	
Penetrating trauma	8 (2.31)	3 (1.95)	5 (2.59)	
Blunt trauma	22 (6.34)	8 (5.19)	14 (7.25)	
Solo bicycle crash	9 (2.59)	9 (5.84)	0 (0)	
Other	13 (3.75)	8 (5.19)	5 (2.59)	
Emergent, n (%)	82 (23.6)	44 (28.6)	38 (19.7)	0.057
Length of Stay, mean ± SD	24.0 ± 130	28.5 ± 187	18.4 ± 47.7	0.516
Disposition, n (%)				
Home	190 (54.6)	93 (62.0)	93 (48.9)	**0.174**
Skilled Nursing Facility	75 (21.6)	26 (17.3)	47 (24.7)	
Acute facility transfer	31 (8.91)	14 (9.33)	16 (8.42)	
Other facility	20 (5.75)	5 (3.33)	14 (7.37)	
In-patient Rehab	17 (4.89)	8 (5.33)	9 (4.74)	
Homeless/Shelter	9 (2.59)	3 (2.00)	6 (3.16)	
AMA	3 (0.86)	1 (0.67)	2 (1.05)	
In-Hospital Death	3 (0.86)	0 (0)	3 (1.58)	
Mal-union, n (%)	3 (0.86)	1 (0.65)	2 (1.04)	0.695
Nonunion, n (%)	10 (2.87)	4 (2.58)	6 (3.11)	0.769
Reoperation, n (%)	60 (17.2)	29 (18.7)	31 (16.1)	0.516
Total Follow-up, mean ± SD	233 ± 385	249 ± 410	217 ± 359	0.445

Note: Bolded *p*-values indicate statistical significance (*p* < 0.05). Abbreviations: ADI: Area Deprivation Index; AMA: Against Medical Advice; DC: Distressed Community; ISS: Injury Severity Score; NDC: Non-Distressed Community; SD: Standard Deviation. ISS, length-of-stay, and disposition comparisons exclude eight patients whose state ADI could not be assigned (NDC *n* = 150; DC *n* = 190); discharge home differed between the groups (OR 0.59, 95% CI: 0.38 to 0.91; *p* = 0.021)

When comparing the demographics between the two cohorts split by socioeconomic distress, patients from DC more frequently had Medi-Cal (28.1%), Medicare (22.9%), California Children’s Services (6.54%), or Veterans Affairs (4.58%; *p* = 0.003), with higher odds of public insurance coverage overall (OR 1.90, 95% CI: 1.22–2.96; *p* = 0.006) (Table 1). Patients from DC also had higher odds of a prior smoking history (OR 2.10, 95% CI: 1.36–3.26) and of a psychiatric history (OR 2.78, 95% CI: 1.39–5.55) (Table 1). Additionally, patients from DC were more frequently injured by motor vehicle crash (35.2%), motorcycle (12.4%), auto vs. pedestrian (14.5%), and blunt (7.25%) or penetrating (2.59%) trauma (*p* = 0.045). Although patients from NDC had a higher rate of emergent orthopaedic injuries, this result was not statistically significant (28.6 vs. 19.7%; *p* = 0.057) (Table 2). Mean ISS did not differ between the groups (18.8 ± 11.9 vs. 19.8 ± 13.2; *p* = 0.435) (Table 2). Patients from DC were less likely to be discharged home than patients from NDC (48.9 vs. 62.0%; OR 0.59, 95% CI: 0.38–0.91; *p* = 0.021), while the overall distribution of discharge disposition did not differ significantly between the groups (*p* = 0.174) (Table 2). However, when comparing clinical outcomes between DC and NDC, we did not find significant differences in the rates of malunion, nonunion, and reoperation. Furthermore, no significant differences in the length of stay or follow-up time were observed.

## Discussion

Polytrauma patients are complex patients in which multiple organ systems are often involved and require intense hospital care and follow-up for patients to have good outcomes after their injuries. Our study focused on how certain social determinants of health can affect their care and outcomes. In our study, we found that patients from a distressed community, which was determined by an ADI > 8, had higher odds of a history of psychiatric illness (OR 2.78) or smoking (OR 2.10) and of public health insurance coverage (OR 1.90), and were less likely to be discharged home (OR 0.59).

This study identified a high rate of psychiatric comorbidities among patients from distressed communities. This is important to note since a previous study which looked at socioeconomic factors regarding mental health treatment found that lower education status was a strong predictor of not receiving treatment and another study noted that psychiatric diagnoses like anxiety and depression are directly correlated with poverty levels [17, 18]. Additionally, other studies have shown that psychiatric diagnoses, specifically depression, are associated with orthopaedic complications and higher rates of noncompliance following discharge [19, 20].

Our study found differences in racial group composition of the two ADI groups (*p* = 0.002), however, we did not find statistically significant difference in ethnicity composition. These results are consistent with other studies that evaluate social determinants of health which found that people living in disadvantaged neighborhoods more frequently identified as non-White or another racial minority and had worse health at baseline compared with those living in non-disadvantaged neighborhoods [21]. When evaluating insurance status, this study showed that patients from distressed communities more frequently had public insurance (OR 1.90, 95% CI: 1.22–2.96). Insurance status has been well documented as a factor for increased mortality, shorter hospital stays, more severe mechanisms of injury, and increased complications in trauma patient [22–25]. Although our study did not have significant findings for differences in length of stays, we did see higher rates of more severe mechanisms of injury like MVC, motorcycle crash, auto vs. pedestrian, and blunt trauma in the DC group. Notably, mean ISS did not differ between the groups (18.8 vs. 19.8; *p* = 0.435), indicating that these differences in mechanism did not translate into greater overall anatomic injury severity in the DC group.

Lastly, our study found a difference between the two groups regarding discharge disposition. Patients from distressed communities were less likely to be discharged home (48.9 vs. 62.0%; OR 0.59, 95% CI: 0.38–0.91), while rates of discharge to skilled nursing facilities, inpatient rehabilitation, shelters, and departures against medical advice did not differ significantly between the groups. Despite this difference in discharge disposition, our study did not find a significant difference in mean follow-up or clinical outcomes, which have been reported in other studies. Jung et al. found that patients in disadvantaged areas who were discharged home and used home health aides had overall worse health outcomes due to use of lower quality home health aides and other factors like lack of financial and social support [21]. This highlights the need for further evaluation of the institutional policies or community factors that are helping support the equivalent clinical outcomes for patients from distressed communities.

Although this study provides valuable insight into a unique trauma patient population, it is not without limitations. As a retrospective study, there is a risk of selection bias, however this was limited by including all patients during the period evaluated. Furthermore, the outcome data are limited because it is difficult to collect long-term follow-up data on polytrauma patients, many of whom receive subsequent care outside the index trauma center, which may limit our ability to find statistically significant differences in long-term outcomes.

## Conclusions

In conclusion, our study found significant differences in polytrauma patient demographics, injury variables, and discharge to home when we used ADI to stratify groups. These findings emphasize the importance of systematic strategies to support clinical care for vulnerable populations. This study did not find statistically significant differences in health outcomes based on socioeconomic status, however more research is needed to identify the impacts of the management strategies at our institution that are supporting equivalent clinical outcomes. Furthermore, we advocate for the use of ADI as a baseline characteristic in orthopaedic clinical research to help standardize clinical outcome comparisons across regions with significantly different populations. Lastly, the increased awareness of the impacts of the social determinants of health is necessary for the improvement of clinical outcomes for underrepresented communities and for the advancing health equity in trauma care delivery.

## Data Availability

The datasets generated and analyzed during the current study are not publicly available in order to protect patient privacy, but are available from the corresponding author on reasonable request and subject to the relevant institutional approvals.
